# Circumcision for HIV Prevention: Failure to Fully Account for Behavioral Risk Compensation

**DOI:** 10.1371/journal.pmed.0040138

**Published:** 2007-03-27

**Authors:** Seth Kalichman, Lisa Eaton, Steven Pinkerton

Three randomized controlled trials (RCTs) of male circumcision (MC) have been halted when interim analyses showed significant reductions in HIV infection among men who received this intervention [1–3]. Modeling suggests that increased MC coverage in southern Africa could prevent as many as 2 million HIV infections over ten years [4]. Moreover, the cost-effectiveness analysis by Kahn et al. recently published in PLoS Medicine indicates that MC could be cost-saving [5]. However, the protection of MC may be partially offset by increased HIV risk behavior, or “risk compensation,” especially reduction in condom use or increases in numbers of sex partners. Risk compensation occurs when individuals adjust their behavior in response to perceived changes in their vulnerability to a disease [6]. Risk compensation may be especially important for MC because avoiding the sexual dissatisfactions of condom use and the desire to have more sex partners are likely to be significant motivations for men to seek circumcision [7]. In South Africa, 73% of men between the ages of 15 and 24 report using condoms during the last time they had sex [8]. It is difficult to imagine a convincing public health message that effectively influences men to undergo circumcision and continue to consistently use condoms.

Circumcised men in the ANRS 1265 trial reported 18% more sexual contacts at follow-up than did uncircumcised men, but no other sexual behavior differences were obtained [1]. However, for ethical reasons all men in MC RCTs receive ongoing risk-reduction counseling and free condoms, which reduces the utility of these trials for estimating the potential behavioral impact of MC when implemented in a natural setting. One model of the potential impact of MC did not take into account risk compensation [4], but noted that “increases in risk-taking behaviour among circumcised men could reduce the benefit of MC.” Based on the 18% difference in sexual contacts for circumcised and uncircumcised men in the ANRS 1265 trial and the assumption that “risk compensation might be higher in a nonresearch program scale-up,” Kahn et al. [5] adjusted the 60% effectiveness estimate obtained in this RCT downward to 50% to reflect a 25% increase in sexual risk behaviors among circumcised men. Although Kahn et al.'s model explicitly incorporated the increased risk of HIV acquisition associated with risk compensation, it did not consider the impact of risk compensation on the HIV transmission risk of HIV-infected circumcised men, or on circumcised men's risk for non-HIV sexually transmitted infections (STIs).

There is no evidence that circumcision increases or decreases the risk of HIV transmission by HIV-infected men. However, risk compensation by HIV-infected circumcised men will substantially increase the risk of transmission to their sex partners. This suggests that, in the short term at least, circumcision would reduce the incidence of HIV among men, but increase the incidence among women, translating to increased prevalence among women, which in turn translates to greater risk to men. Epidemiological models of MC should take this dynamic into account.

Countless studies have shown that ulcerative and non-ulcerative STIs account for at least some of the rapid increases in HIV transmission in southern Africa [9]. Non-HIV STIs are associated with a 2- to 5-fold increase in HIV transmission risk in countries with low and high rates of MC [9]. In areas with prevalent STIs, the relative increase in men's STI-associated HIV risk can be as high as 60% to 340% [10]. Circumcision likely reduces the risk of acquiring a non-HIV STI and may be partially responsible for the decreased HIV risk observed in circumcision RCTs [1]. Nevertheless, the failure of models to account for increased STI risk due to risk compensation likely inflates estimates of averted HIV infections. Estimates of HIV risks resulting from increased exposure to STIs that coincide with reductions in condom use have been included in previous models of the cost-effectiveness of HIV prevention interventions [11] and should be included in MC models.
